# The relationship of CDK18 expression in breast cancer to clinicopathological parameters and therapeutic response

**DOI:** 10.18632/oncotarget.25686

**Published:** 2018-06-29

**Authors:** Giancarlo Barone, Arvind Arora, Anil Ganesh, Tarek Abdel-Fatah, Paul Moseley, Reem Ali, Stephen YT Chan, Constantinos Savva, Kristina Schiavone, Natasha Carmell, Katie N. Myers, Emad A. Rakha, Srinivasan Madhusudan, Spencer J. Collis

**Affiliations:** ^1^ Sheffield Institute for Nucleic Acids (SInFoNiA), Academic Unit of Molecular Oncology, Department of Oncology and Metabolism, University of Sheffield Medical School, Sheffield, UK; ^2^ Academic Unit of Oncology, Division of Cancer and Stem Cells, School of Medicine, University of Nottingham, Nottingham, UK

**Keywords:** CDK18, breast cancer, replication stress, chemotherapy, cyclin-dependent kinase

## Abstract

**Background:**

Cyclin-Dependent Kinases (CDKs) are established anti-cancer drug targets and a new generation of CDK inhibitors are providing clinical benefits to a sub-set of breast cancer patients. We have recently shown that human CDK18 promotes efficient cellular responses to replication stress. In the current study, we have investigated the clinicopathological and functional significance of CDK18 expression levels in breast cancers.

**Results:**

High CDK18 protein expression was associated with a triple negative and basal-like phenotype (*p* = 0.021 and 0.027 respectively) as well as improved patient survival, which was particularly significant in ER negative breast cancers (*n* = 594, Log Rank 6.724, *p* = 0.01) and those treated with chemotherapy (*n* = 270, Log Rank 4.575, *p* = 0.03). In agreement with these clinical findings, breast cancer cells genetically manipulated using a dCRISPR approach to express high levels of endogenous CDK18 exhibited an increased sensitivity to replication stress-inducing chemotherapeutic agents, as a consequence to defective replication stress signalling at the molecular level.

**Conclusions:**

These data reveal that CDK18 protein levels may predict breast cancer disease progression and response to chemotherapy, and provide further rationale for potential targeting of CDK18 as part of novel anti-cancer strategies for human cancers.

**Materials and Methods:**

CDK18 protein expression was evaluated in 1650 breast cancers and correlated to clinicopathological parameters and survival outcomes. Similar analyses were carried out for genetic and transcriptomic changes in CDK18 within several publically available breast cancer cohorts. Additionally, we used a deactivated CRISPR/Cas9 approach (dCRISPR) to elucidate the molecular consequences of heightened endogenous CDK18 expression within breast cancer cells.

## INTRODUCTION

Breast cancer is the most prevalent cancer in women worldwide, and the second most common cancer overall behind lung cancer. In the UK, around 50,000 new breast cancer cases are diagnosed each year, and breast cancer accounts for around 12,000 deaths each year [1]. Many breast cancers do not respond well to traditional chemotherapy, and triple-negative tumours are particularly difficult to successfully treat. As such, new approaches and agents are currently being sought for the treatment for these tumours [1, 2]. Cyclin dependent kinases (CDKs) are an evolutionary conserved family of over 20 distinct Ser/Thr kinases that regulate both normal cycle progression and various stress-induced cell cycle checkpoints [3]. The importance of CDKs in controlling cellular growth/proliferation is highlighted by the high prevalence of dysfunction and/or aberrant expression of this family of proteins within human cancers [4]. As such, the development of small molecule CDK inhibitors (CDKi) has been the focus of many research groups and the pharmaceutical industry over the past 20 years [5]. Unfortunately, specific and selective targeting of individual CDKs has proved difficult, giving rise to non-specific activity and detrimental toxicities, which has hindered wide-scale use of CDKi within the clinic. However, recent advancements in the development of the next generation of specific CDKi combined with the emergence of more reliable and informative biomarkers have reinvigorated the field of CDKi [5]. This is especially true for breast cancer, where the CDK4/6 inhibitors Palbociclib/Ibrance (Pfizer), Ribociclib/Kisqali (Novartis & Astex) and Abemaciclib/Verzenio (Eli Lilly) were recently licensed for use in combination with hormonal-based therapies for the management of ER+/HER2-advanced metastatic breast cancers due to the significant progression-free survival benefits it offers these patients [6–8]. These recent discoveries have led to an increased interest in targeting other CDKs within breast tumours [9].

We recently reported functional characterisation of the human CDK family member CDK18, demonstrating that it promotes robust activation of ATR-mediated signalling in response to replication stress [10]. Elevated replication stress is prevalent in a high proportion of human cancers, which is often driven by the activation of oncogenes [11, 12]. As such, targeting of factors that lead to catastrophic levels of replication stress within cancer cells is an active area of both pre-clinical and clinical research [13–15]. Due to its cellular functions, CDK18 therefore represents a potential novel anti-cancer drug target, both as a mono-therapeutic approach in cancers with high levels of replication stress, and as part of combination approaches with chemotherapeutic agents that induce high levels of replication stress and/or DNA damage. Here we report our analysis of differential CDK18 mRNA and protein expression levels in relation to clinicopathological parameters within human breast cancers utilising large independent breast cancer clinical cohorts. We also report our phenotypic characterisation of deactivated dCRISPR-mediated genetically engineered breast cancer cell models that over express the endogenous CDK18 gene, and how these phenotypes may relate to our findings in breast cancer patients.

## RESULTS

### Genomic and transcriptomic analysis of CDK18 in human breast cancers

**Figure 1:**
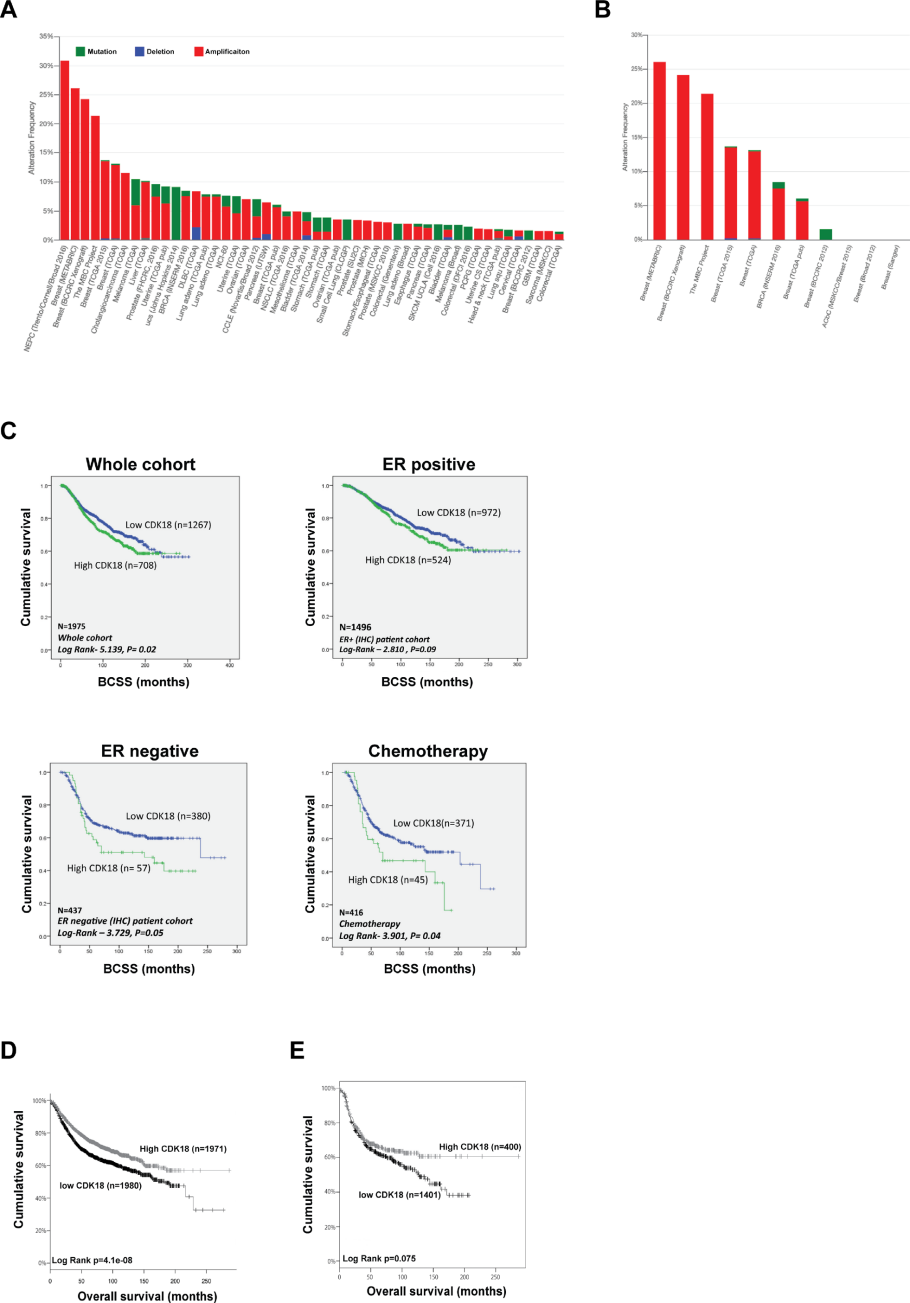
Genetic and transcriptomic analysis of CDK18 in breast cancer cohorts (**A**) Prevalence of CDK18 amplification (red; mainly due to copy number variance gains), deletion (blue) and mutations (green) across human cancers (derived from cBioPortal; http://www.cbioportal.org/). Pink circles under the bar chart represent breast cancer cohorts, which show a high prevalence for CDK18 amplification. (**B**) CDK18 amplification from the cBioPortal data stratified for breast cancer cohorts, showing high frequency of CDK18 CNV gains across multiple breast cancer cohorts (pink circles). (**C**) Kaplan–Meier survival curves derived from analysis of the METABRIC dataset of around 1980 breast cancer patients, plotted for CDK18 mRNA expression against breast cancer-specific survival (BCSS) and stratified as indicated above each graph. The chemotherapy data was derived from patients whose tumours were treated with the replication stress-inducing agents 5-FU, methotrexate and/or cyclophosphamide. (**D**) Kaplan–Meier survival curves of CDK18 mRNA expression (above or below median mRNA expression levels across the cohorts) derived from combined TGCA and EGA breast cancer cohorts (KMplotter; [45]; http://kmplot.com/analysis/index.php?p=service). (**E**) Same as in (**D**), but stratified for ER- tumours.

As part of our interest in a potential role for CDK18 in cancer biology and putative therapeutic target, we interrogated CDK18 genomic alterations in a range of published cancer genome databases. From 162 studies representing over 45,000 samples, CDK18 gene amplification (due to copy number variance gain) was vastly more prevalent than deletions or mutations, with 4 out of the top 5 incidences of reported CDK18 amplification occurring in breast cancer cohorts (Figure 1A). Indeed, CDK18 amplification was prevalent in the majority of all the reported breast cancer cohorts (representing ~6000 samples), ranging from ~5–26%, with an average amplification of 11.3% (Figure 1B). Based on these findings, we next investigated whether alterations in CDK18 at the transcriptomic level within breast cancer cohorts might be associated with various aspects of tumour biology and/or patient prognosis. As the largest CDK18 gene amplification occurs within METABRIC dataset (Figure 1A and 1B), which is the most comprehensive breast cancer cohort for which detailed clinicopathological information is known [16], we further interrogated CDK18 mRNA expression levels within this cohort. Overall, there was no significant correlation between CDK18 mRNA expression levels and clinicopathological parameters (Supplementary Table 1). However, elevated CDK18 mRNA expression (above median) was associated with reduced patient survival across the whole cohort (*n* = 1975, Log Rank -5.139, *p* = 0.02), which was also true for ER- tumours (*n* = 437, Log Rank –3.729, *p* = 0.05), but not for ER+ tumours (Figure 1C). Strikingly, breast cancers exhibiting elevated CDK18 mRNA expression were associated with a poorer response to the commonly used replication stress-inducing chemotherapeutic agents 5-FU, cyclophosphamide and methotrexate (*n* = 416, Log Rank -3.901, *p* = 0.04; Figure 1C). This is consistent with our recent findings demonstrating that CDK18 promotes robust cellular responses to chemically induced replication stress [10]. However, in contrast to these findings, analysis of combined EGA and TCGA breast cancer samples (KM Plotter) suggests that high (above median) rather than low levels of CDK18 mRNA expression are associated with better patient survival (*n* = 3951, Log Rank *P* = 4.1e^–8^; Figure 1D), with a similar trend for ER- tumours (*n* = 801, HR = 0.81, Log Rank *P* = 0.075; Figure 1E), but not ER+ tumours (*n* = 2061, HR = 1, Log Rank *P* = 0.98; data not shown). Although gene amplification often leads to a subsequent increased mRNA and/or protein expression, it is commonly accepted that this is not always the case [17]. This is in part due to the genomic loci of the amplification, the complex compound genetic changes that occur within tumours, and the numerous epigenetic regulatory mechanisms that can negate gene amplification at both the mRNA and protein level [17]. Overall, these data suggest that subsequent CDK18 protein expression levels and/or cellular activity might be important for aspects breast cancer biology and treatment outcomes.

### CDK18 protein expression in human breast cancers and clinicopathological associations

The associations between CDK18 amplification and/or mRNA expression levels with breast cancer patient survival prompted us to investigate CDK18 protein expression within breast cancers in relation to clinicopathological phenotypes. To facilitate quantitative immunohistochemical studies, FFPE sections of breast cancer cells transfected with either non-targeting control siRNA or previously validated CDK18 siRNA [10] were used to optimise IHC staining conditions (Supplementary Figure 1A and 1B). To validate the optimised CDK18 antibody conditions on human tissue sections, CDK18 immunohistochemical staining was assessed in commercial breast cancer tissue microarrays comprising of over 360 core biopsies of various cancer lineages, stage and grade, as well as normal healthy breast tissue and cancer adjacent controls (Supplementary Figure 1C). Consistent with our localisation studies in mammalian cell lines [10], and that many DDR proteins reside and function within both the cytoplasm and nucleus, CDK18 was expressed in both the nucleus and cytoplasm of breast tissue (Supplementary Figure 1A–1C). Interestingly, increased CDK18 expression was associated with cancerous tissue compared to healthy and non-cancerous adjacent tissue (OR = 9.655, *p* =< 0.001; Supplementary Figure 1C and 1D), although CDK18 expression did not continue to increase beyond grade 2 tumours within these tissue microarrays (Supplementary Figure 1D). However, these TMAs only contained 23 grade 3 tumours compared with 63 grade 1 and 170 grade 2 tumours.

**Figure 2:**
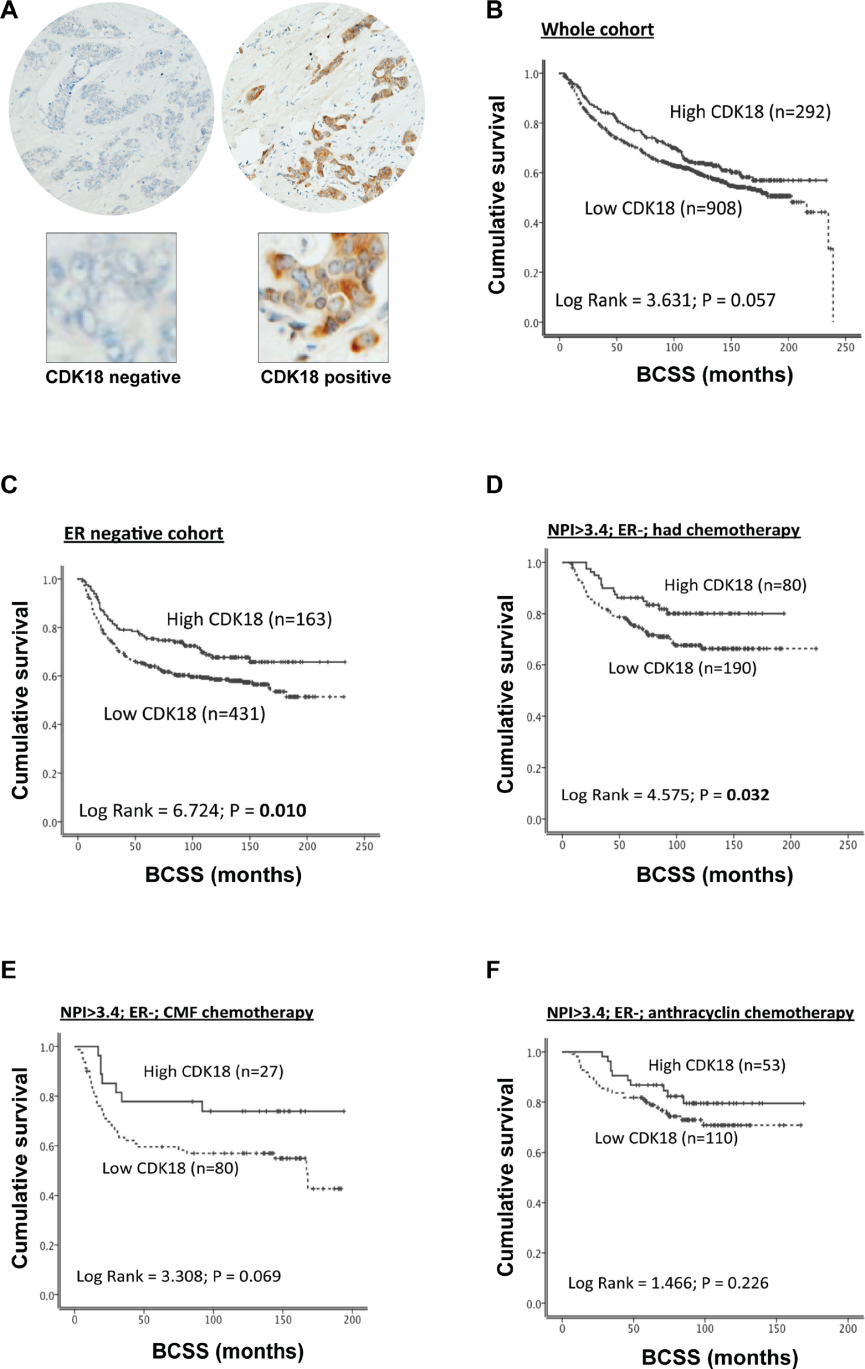
Analysis of CDK18 protein expression in the Nottingham Tenovus breast cancer cohort (**A**) Examples of negative control staining (left panel) and a CDK18 positively stained core breast cancer TMA (right panel), with enlarged images below highlighting the high prevalence of cytoplasmic CDK18 expression. (**B**) Kaplan–Meier survival curves for CDK18 protein expression (above or below median) plotted against breast cancer specific survival in the whole Nottingham Tenovus breast cancer cohort. (**C**–**F**) Kaplan–Meier survival curves for CDK18 protein expression (above or below median) plotted against breast cancer specific survival for the ER- tumours within the Nottingham Tenovus breast cancer cohort stratified using the indicated clinicopathological parameters. CMF; tumours treated with the combination cyclophosphamide, methotrexate and 5-FU clinical chemotherapeutic regime.

Using these optimised IHC conditions, we proceeded to investigate CDK18 protein expression in the Nottingham Tenovus series of 1650 breast cancers for which detailed clinicopathological parameters and various molecular markers have been previously defined [18–21]. Similar to the commercial breast cancer TMAs, we observed nuclear as well as cytoplasmic staining of CDK18, however, nuclear expression was rare and cytoplasmic expression was more common within these samples (Figure 2A). High cytoplasmic CDK18 protein expression was associated with triple negative and basal-like phenotype (*p* = 0.021 and *p* = 0.027 respectively; Table 1), and low levels of CDK18 expression were strongly correlated with HER2 overexpression (*p* < 0.001; Table 1). Furthermore, high cytoplasmic CDK18 expression was associated with markers of DNA repair (Table 2), including high ATR (*p* = 0.005), high APE1 (*p* < 0.001), high Polβ (*p* < 0.001) and high DNA-PK_cs_ (*p* < 0.001). Similarly, high cytoplasmic CDK18 expression was also linked to markers of cell cycle regulation (Table 2), such as phosphorylated CHK1 (pCHK1; *p* = 0.001), high p16 (*p* = 0.018), high CHK2 (*p* = 0.002), high CDK1 (*p* = 0.004) and high MDM2 (*p* = 0.047). In terms of survival outcomes, low CDK18 protein expression was associated with poorer patient survival (*n* = 1200, Log Rank 3.631, *p* = 0.06; Figure 2B), which was particularly significant within the ER- cohort, where high cytoplasmic CDK18 was associated with an overall better survival (*p* = 0.010; Figure 2C), and in patients that received chemotherapy (*p* = 0.032; Figure 2D). In the sub-group that received CMF based chemotherapy, there was a similar trend with better survival in patients with high cytoplasmic CDK18 expressing tumours (*p* = 0.07; Figure 2E), but not in patients who received anthracycline-based chemotherapy (Figure 2F). Consistent with CDK18 mRNA expression, CDK18 protein expression did not influence survival within ER+ tumours, (Supplementary Figure 2A–2C). Collectively, these data suggest that CDK18 expression could potentially predict response to chemotherapeutic agents, and may influence certain aspects of breast cancer biology.

**Table 1:** Association of CDK18 protein expression with aggressive tumour genotypes/phenotypes in the Nottingham Tenovus breast cancer cohort

Variable	CDK18 (cytoplasmic) Protein Expression		*P*- value
	Low *N* (%)	High *N* (%)	
**Mitotic Index**			
M1 (low; mitoses < 10)	308 (33.8)	88 (30.1)	0.470
M2 (medium; mitoses 10–18)	172 (18.9)	61 (20.9)	
M3 (high; mitosis > 18)	430 (47.3)	143 (49.0)	
**Her2 overexpression**			
No	775 (86.3)	272 (95.1)	**5.0 × 10^–5^**
Yes	123 (13.7)	14 (4.9)	
**Triple Negative Phenotype**			
No	737 (82.4)	219 (76.3)	**0.021**
Yes	157 (17.6)	68 (23.7)	
**Basal Like Phenotype**			
No	765 (88.9)	233 (83.8)	**0.027**
Yes	96 (11.1)	45 (16.2)	
**Cytokeratin 6 (CK6)**			
Negative	651 (84.2)	197 (82.1)	0.434
Positive	122 (15.8)	43 (17.9)	
**Cytokeratin 14 (CK14)**			
Negative	667 (86.7)	215 (90.0)	0.188
Positive	102 (13.3)	24 (10.0)	
**Cytokeratin 18 (CK18)**			
Negative	73 (10.1)	27 (12.4)	0.330
Positive	652 (89.9)	191 (87.6)	
**Cytokeratin 19 (CK19)**			
Negative	49 (6.4)	13 (5.4)	0.576
Positive	712 (93.6)	226 (94.6)	
**ATF2**			
Low	351 (50.8)	110 (48.9)	0.619
High	340 (49.2)	115 (51.5)	

HER2: human epidermal growth factor receptor 2; CK: cytokeratin; Basal-like: ER-, HER2 and positive expression of either CK5/6, CK14 or EGFR; Triple negative: ER-/PgR-/HER2-.

**Table 2:** Association of CDK18 protein expression with protein expression of the indicated DNA repair factor, cell cycle or apoptotic regulator within the Nottingham Tenovus breast cancer cohort

Variable	CDK18 (cytoplasmic) Protein Expression		*P*- value
	Low *N* (%)	High *N* (%)	
**BRCA1**			
Absent	135 (20.8)	34 (16.3)	0.149
Normal	513 (79.2)	175 (83.7)	
**XRCC1**			
Low	117 (17.7)	27 (12.4)	0.065
High	543 (82.3)	191 (87.6)	
**FEN1**			
Low	469 (74.9)	134 (69.1)	0.107
High	157 (25.1)	60 (30.9)	
**SMUG1**			
Low	241 (39.6)	69 (35.4)	0.288
High	367 (60.4	126 (64.6)	
**APE1**			
Low	416 (54.6)	92 (36.7)	**1.0 × 10^–6^**
High	346 (45.4)	159 (63.3)	
**Pol β**			
Low	344 (43.2)	73 (27.2)	**4.0 × 10^–6^**
High	453 (56.8)	195 (72.8)	
**ATR**			
Low	521 (68.8)	152 (59.1)	**0.005**
High	236 (31.2)	105 (40.9)	
**ATM**			
Low	296 (52.9)	92 (51.4)	0.733
High	264 (47.1)	87 (48.6)	
**DNA-PKcs**			
Low	285 (39.3)	56 (23.6)	**1.3 × 10^–5^**
High	441 (60.7)	181 (76.4)	
**P16**			
Low	544 (88.0)	167 (81.5)	**0.018**
High	74 (12.0)	38 (18.5)	
**P21**			
Low	369 (55.8)	113 (56.5)	0.866
High	292 (44.2)	87 (43.5)	
**MIB1**			
Low	339 (45.9)	96 (39.3)	0.075
High	400 (54.1)	148 (60.7)	
**P53**			
Low expression	595 (79.4)	176 (75.5)	0.205
High expression	154 (20.6)	57 (24.5)	
**Bcl-2**			
Negative	309 (37.5)	84 (33.2)	0.214
Positive	515 (62.5)	169 (66.8)	
**TOP2A**			
Low	310 (49.1)	82 (36.9)	**0.002**
Overexpression	322 (50.9)	140 (63.1)	
**pChk1 (Nuclear)**			
Low	801 (87.5)	220 (74.3)	**1.0 × 10^–6^**
High	114 (12.5)	76 (25.7)	
**pChk1 (Cytoplasmic)**			
Low	314 (34.3)	72 (24.3)	**0.001**
High	601 (65.7)	224 (75.7)	
**Chk2**			
Low	335 (51.6)	70 (33.5)	**5.0 × 10^-6^**
High	314 (48.4)	139 (66.5)	
**Bax**			
Low	371 (70.3)	111 (66.1)	0.305
High	157 (29.7)	57 (33.9)	
**CDK1**			
Low	414 (72.9)	114 (61.6)	**0.004**
High	154 (27.1)	71 (38.4)	
**MDM2**			
Low	495 (78.0)	140 (71.1)	**0.047**
Overexpression	140 (22.0)	57 (28.9)	

### Heightened expression of endogenous CDK18 in breast cancer cells leads to defective replication stress signalling

**Figure 3:**
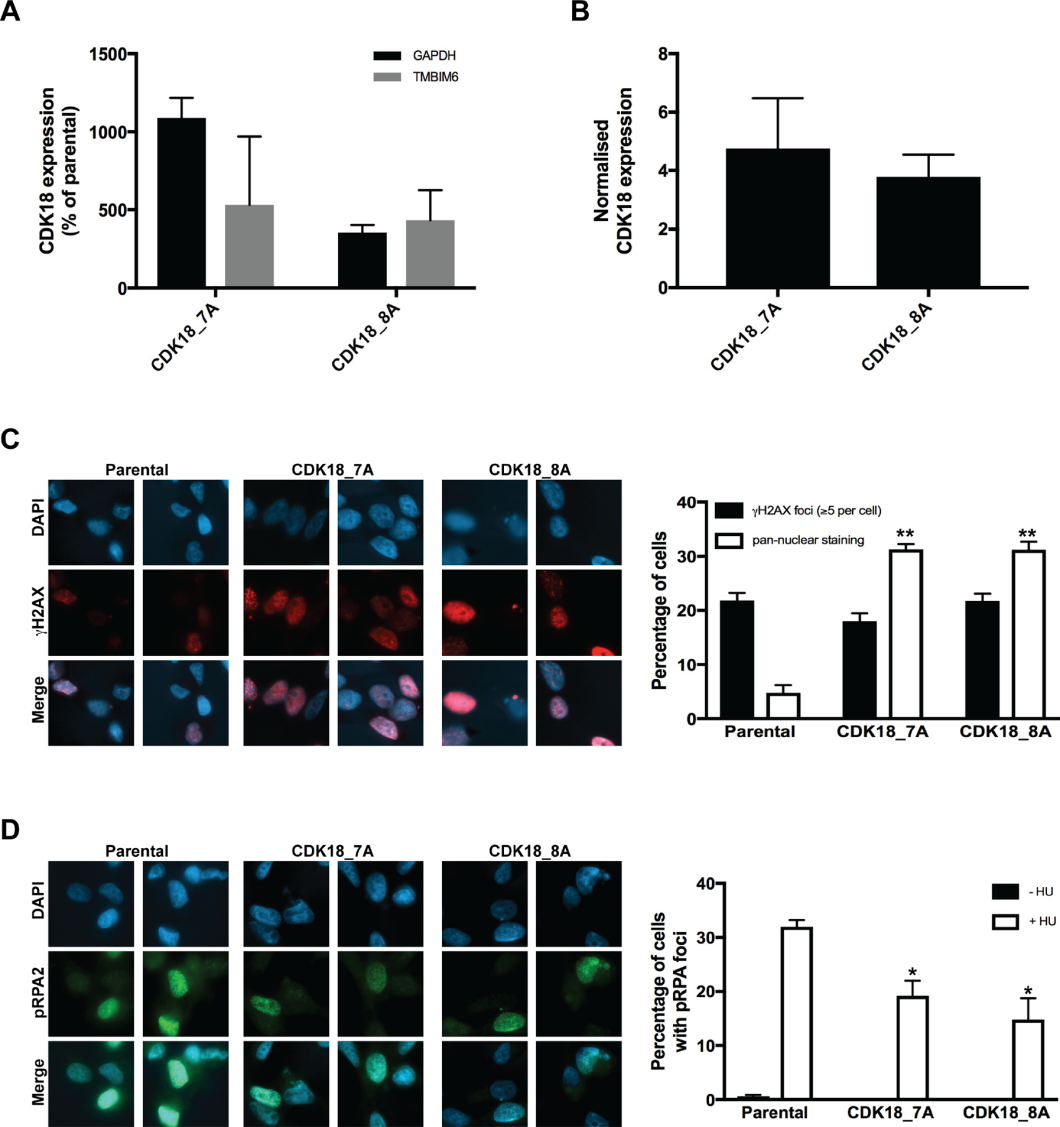
Deactivated CRISPR/Cas9 mediated activation of endogenous CDK18 leads to replication stress response defects in breast cancer cells (**A**) RT-PCR analysis of endogenous CDK18 mRNA expression levels in two independently derived deactivated CRISPR/Cas9 CDK18 activation MDA-MB-231 clones 7A and 8A. CDK18 mRNA levels were normalised to either GAPDH or TMBIM6 reference genes as indicated. The data shown represents the mean mRNA expression compared to parental MDA-MB-231 cells with respective SEMs derived from three independent experiments. (**B**) Normalised CDK18 protein expression levels in CRISPR/Cas9 CDK18 activation MDA-MB-231 clones 7A and 8A compared to parental MDA-MB-231 cells. Data shown represents the quantified mean derived from four independent western blot experiments with their respective SEM. (**C**) Left panel; representative images of immunofluorescence staining of γH2AX in parental MDA-MB-231 cells and CDK18 activation clones 7A and 8A as indicated. Right panel; quantification of γH2AX nuclear foci and pan-nuclear γH2AX staining in the indicated cell lines. Data shown represents the means derived from three independent experiments with their respective SEMs (^*^p ≤ 0.05 and ^**^p ≤ 0.01 compared to parental MDA-MB-231 cells). (**D**) Same as in C, but for pRPA2 (Thr21) nuclear foci in untreated and HU treated (3mM, 4hrs) cell lines as indicated.

**Figure 4:**
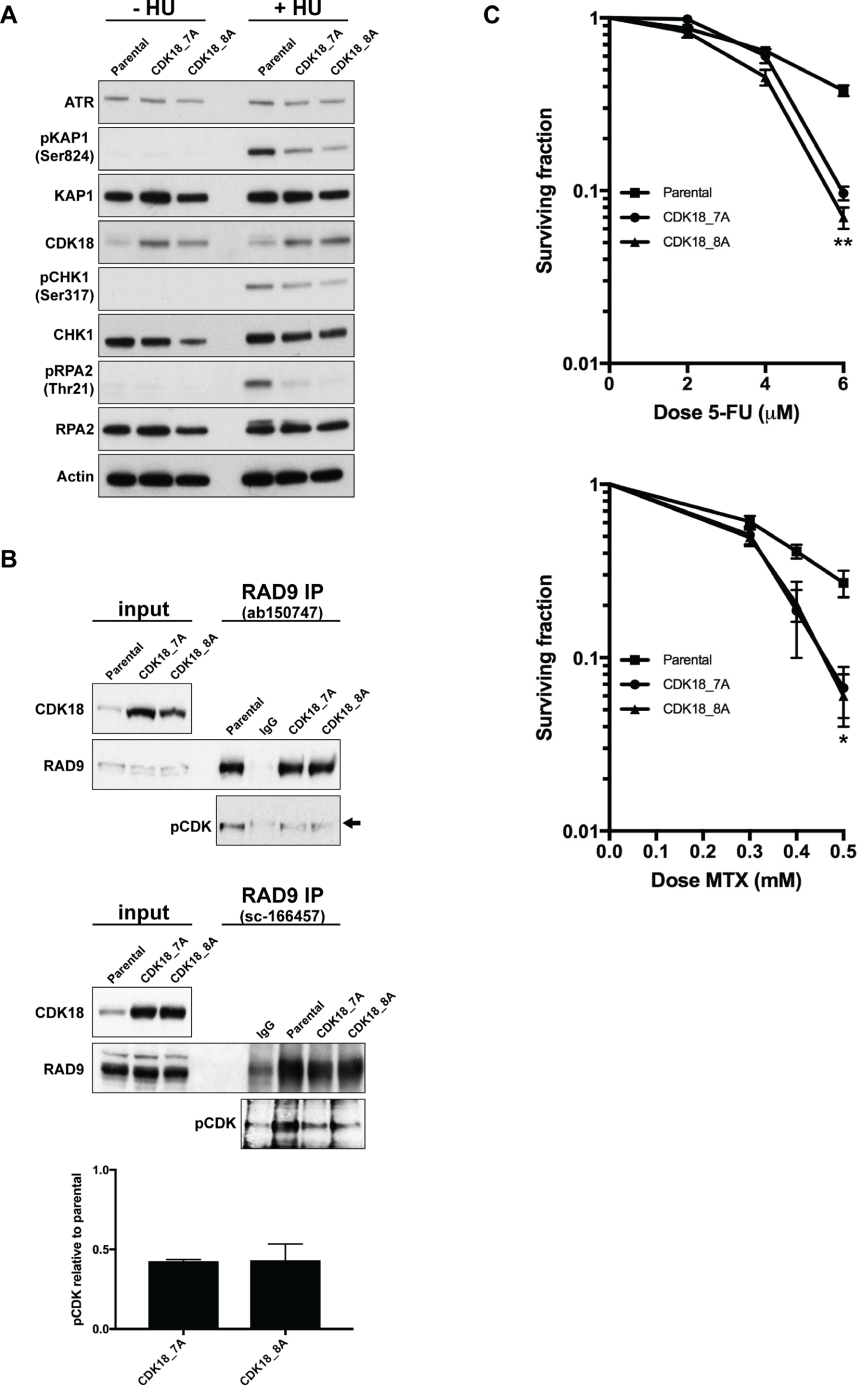
Heightened expression of endogenous CDK18 disrupts replication stress signalling and confers an increased sensitivity to replication stress-inducing agents (**A**) Representative western blots for the indicated proteins in untreated and HU-treated parental and CDK18 activation MDA-MB-231 clones. (**B**) Representative western blots of endogenous RAD9 IPs from parental and CDK18 activation clones using two separate human RAD9 mouse monoclonal antibodies (upper and middle panels as indicated). Equal amounts of immunoprecipitates were probed with either RAD9 (rabbit polyclonal) or phospho-CDK substrate (K/HpSP motif; pCDK) antibodies as indicated. Inputs demonstrate heightened CDK18 expression in the activation clones and comparable RAD9 expression. IgG was used as a negative control for non-specific binding which can be seen as a faint band migrating at a slightly higher molecular weight to RAD9 (black arrow). Levels of pCDK in the RAD9 IPs were quantified from these data by normalising to the relative amount of immunoprecipitated RAD9 within each cell population. The lower panel shows the quantified average pCDK levels in the indicated cell populations with their respective SEMs. (**C**) Clonogenic survival curves for 5-FU (left panel) and Methotrexate (MTX) treated (right panel) MDA-MB-231 cell lines as indicated. Data shown represents the mean derived from at least three independent experiments with their respective SEMs (^*^p ≤ 0.05 and ^**^p ≤ 0.01 compared to parental MDA-MB-231 cells at the same drug dose).

To further understand our findings in the clinical cohorts, we next investigated the consequences of increased CDK18 expression at a molecular level using breast cancer cell culture models. To achieve this, we employed a deactivated CRISPR/Cas9 system to up-regulate the endogenous CDK18 promoter in MDA-MB-231 breast cancer cells due to its phenotypic comparison with difficult to treat breast cancers e.g. triple negative status and intermediate response to chemotherapy [22]. We also attempted to generate similar CDK18 cell models in the normal breast epithelial cell line MCF-10A, but were unable to generate viable clones (data not shown). Using this approach, we generated 2 separate sub-clones that exhibited between 5–10 fold increased expression of endogenous CDK18 mRNA over parental MDA-MB-231 cells (Figure 3A), resulting in an ~4-fold increase in endogenous CDK18 protein expression (Figures 3B, 4A and 4B). As we have recently shown that cells with depleted levels of CDK18 exhibit elevated levels of DNA damage due to defects in replication stress signalling [10], we assessed endogenous DNA damage levels in these CDK18 activation clones using the DNA damage marker γH2AX. In contrast to CDK18-depleted cells [10], we did not observe any differences in nuclear foci γH2AX between the activation clones and parental cells (Figure 3C), however, the activation clones exhibited a significant increase in pan-nuclear γH2AX staining (Figure 3C), which is often associated with aberrant replication processes [10, 23]. Consistent with these findings, the CDK18 activation clones exhibited reduced phosphorylation of the replication stress marker RPA2 in response to chemically induced replication stress (Figure 3D).

These data suggest that similar to what we have recently reported for CDK18-depleted cells [10]; heightened expression of endogenous CDK18 compromises ATR-mediated replication stress signalling. Indeed, we found that ATR activation and subsequent phosphorylation of key downstream targets in response to replication stress was abrogated in both CDK18-activaiton clones compared with parental MDA-MB-231 cells (Figure 4A). Our previous studies have revealed that CDK18 physically interacts with RAD9 [10], a key component of the 9-1-1 replication stress signalling complex, and promotes its phosphorylation on proline-directed CDK sites that are critical for establishing an efficient cellular response to replication stress [24–26]. Consistent with defective replication stress signalling, MDA-MB-231 cells exhibiting heightened levels of endogenous CDK18 also display reduced phosphorylation of RAD9 at putative CDK sites (Figure 4B), which again phenocopies that of CDK18-deificent cells [10]. This highlights a likely molecular mechanism to the underlying ATR signalling defect observed in these cells, particularly as significant differences in either cell growth or cell cycle were not observed in the CDK18 activation clones compared to parental cells (Supplementary Figure 2D and 2E). Finally, we assessed the phenotypic consequences of the reduced replication stress signalling in the CDK18 activation clones by carrying out clonogenic survival assays using the breast cancer chemotherapeutic agents 5-FU and Methotrexate. As shown in Figure 4C, both CDK18 activation clones exhibited an increased sensitivity to these replication stress inducing agents compared with parental MDA-MB-231 cells (Figure 4C). Overall, the data from the CDK18 activation clones is consistent with our findings in breast cancer clinical cohorts, where elevated levels of endogenous CDK18 protein expression confers an improved repose to chemotherapeutic agents that induce high levels of replication stress.

## DISCUSSION

Our findings reveal that amplification of the CDK18 gene locus and CNV gains are widespread across human cancers, with a particularly high prevalence in breast cancers. We also find that breast cancers exhibit higher levels of both CDK18 mRNA and protein compared with normal breast tissue. However, it is difficult to disseminate if these are beneficial alterations actively selected for during tumourigenesis, or merely a consequence of other genetic changes that have occurred through the development of these tumours. If alterations in CDK18 expression are indeed beneficial to tumour development, our data suggest that this may be a complex relationship in breast cancer. For example, we observed opposite survival associations between heightened CDK18 mRNA and protein expression in two large breast cancer cohorts (>1600 patients each), although such discrepancies may in part due to the fact that mRNA and protein expression levels often do not correlate. Indeed, analysis of a sub-population of 86 samples within the Nottingham Tenovus breast cancer cohort for which both CDK18 protein and mRNA expression levels were determined, revealed no correlation between CDK18 mRNA and protein expression levels (Supplementary Figure 2F).

One possible way in which differential CDK18 expression may arise in human tumours is as a result of elevated replication stress within the cancerous cells. It is well established that tumours often possess high levels of replication stress due to the activation of oncogenes that can drive genetic alterations through myriad different mechanisms [11, 12, 27–30]. Cellular DNA damage response mechanisms have evolved to act as a barrier to the potential pro-mutagenic increase in DNA damage that can arise through heightened replication stress, and often exhibit differential expression/activity in human tumours [11, 27, 28, 31, 32]. Additionally, previous work from several groups has suggested that specific thresholds of replication stress may exists within different tumour populations that could exert important biological consequences for tumour development and growth and impact on further mutations/genetic alterations that facilitate tumour development [11, 12, 14, 30, 33]. Intriguingly, a recent report identified CNV gains and increased CDK18 mRNA expression in several distinct tumour populations from within a diffuse intrinsic pontine glioma, suggesting a selection preference for such genetic alterations to CDK18 expression during tumour development [34].

Given that CDK18 functions to facilitate robust replication stress signalling [10], any elevated levels of CDK18 in human cancers may simply be a consequence of heightened levels of replication stress and such increased levels of CDK18 could interfere with the finely controlled feedback regulation mechanisms controlling replication stress signalling, ultimately leading to a dominant-negative type disruption to CDK18's normal cellular functions. As such, CDK18 expression levels may provide more significant biological roles in different tumour populations depending on the underlying levels of replication stress and functional status of the various DNA damage response & cell cycle checkpoint machinery. Indeed, we found that CDK18 protein expression was significantly correlated with expression levels of DNA repair factors and cell cycle checkpoint regulators within human breast cancer samples. Specifically, we found that high levels of CDK18 protein expression were associated with high levels of phosphorylated CHK1, which is an established marker of increased replication stress [14]. Consistent with this finding, CDK18 protein levels were also associated with high ATR protein expression; the key kinase that acts upstream of CHK1 to regulate cellular responses to replication stress [35]. Furthermore, we found that high levels of CDK18 were also associated with high levels of TOPBP1 and DNA-PK_cs_, which represent both an established and newly realised factor in cellular responses to replication stress within human cells [36, 37]. It would therefore be interesting to further investigate potential relationships between endogenous levels of replication stress and CDK18 expression in other cancer cohorts. The most established cellular marker of replication stress is phosphorylated RPA2 (pRPA), but to our knowledge, pRPA levels have not been assessed in large cancer patient cohorts, although some initial IHC findings with pRPA2 antibodies are starting to emerge [33], which may help facilitate such future studies.

Within our study, we also find that the association between CDK18 mRNA or protein expression and patient survival is complex within the context of ER status. Our original findings in the METABRIC suggested that high mRNA levels of CDK18 were associated with poor survival, which was also true for in ER-, but not ER+ tumours. However, subsequent analysis of CDK18 protein levels in the Nottingham Tenovus breast cancer cohort revealed better survival in ER- tumours that have higher than median levels of CDK18, which is also true in ER- MDA-MB-231 breast cancer cell lines overexpressing endogenous CDK18 when treated with chemotherapeutic agents. Functional links between the DNA damage response and hormone receptor signalling pathways have been previously reported [2, 38, 39], although the mechanisms by which these two pathways interact have not been fully elucidated [38]. Recent pre-clinical and clinical studies have demonstrated the advantage of inhibiting CDK4/6 driven proliferation in advanced ER+ tumours [6, 7]. Therefore, targeting of other CDKs in cancers with specific genetic backgrounds, phenotypic traits and/or ER status may yield equally promising results [3, 5, 9, 40]. Indeed, CDK18's role in facilitating efficient cellular responses to replication stress make it an attractive therapeutic target in many cancers, including in breast cancer where strong associations between oestrogen receptor signalling, cell proliferation and replication stress are emerging [2, 38, 39]. Our data therefore add to the growing interest in the role of CDKs within breast cancer biology and clinical management strategies, and suggest that further study of CDK18 within other cancers is warranted.

## MATERIALS AND METHODS

### Cell culture and generation of CRISPR/deactivated Cas9 activation clones

MDA-MB-231 and MCF-7 breast cancer cells were maintained as adherent monolayer cultures in DMEM media containing 10% FBS at 37° C in a humidified atmosphere of 5% CO_2_. Stable CDK18 CRISPR/deactivation Cass9 CDK18 activation clones were created using the standard Santa Cruz Biotechnology protocol. Briefly, 1 × 10^5^ cells were seeded into 1 well of a 6 well plate. The following day, 1 ml of media was replaced with 1 ml of fresh FCS free media supplemented with 5 ug/ml of polybrene (SCBT sc-134220) and 20 ul of CDK18 lentiviral particles (SCBT SC-422147). The following day, growth media was replaced with fresh media supplemented with 10% FCS, 2 ug/ml Puromycin (SBCT sc-108071), 300 ug/ml Hygromycin (SBCT sc-29067) and 2 ug/ml Blastacidin (SBCT sc-495389). On day 5, 100 cells were seeded into one 10 cm plate with continued selection. These were left for a further 10 days and individual clones picked, sub-cultured and analysed by RT-PCR and western blotting to confirm activation of the endogenous CDK18 gene promoter.

### Immunofluorescence

1.2 × 10^3^ cells were seeded onto 200 mm glass cover slips in 6 well plates and incubated for 24 hrs for the cells to adhere. Following appropriate siRNA and/or drug treatments, cells were fixed in either ice-cold 100% methanol or 4% PFA for 10 minutes (depending on the antibody used). Coverslips were washed briefly in PBS and extracted with 3% BSA, 0.2 % Triton-X100 dissolved in PBS for 30 minutes, followed by a further brief wash in PBS. Antibodies were diluted in PBS containing 1% BSA at optimised concentrations and a 100 μl aliquot added to each cover slip for 1 hour. This was followed by 3 × 5 minutes washes in PBS. Secondary Alexa-Fluor antibodies (1:500) and DAPI (1 μg/ml) were diluted in PBS containing 1% BSA and a 100 μl aliquot added to each cover slip for 1 hour. This was followed by 3 × 5 minutes washes. Processed coverslips were mounted onto glass slides with 10 μl Shandon immuno-mount (Thermo). Microtubule array assays were carried out as previously described [41]. All immunofluorescence images were captured on a Nikon Eclipse T200 inverted microscope (Melville), equipped with a Hamamatsu Orca ER camera, a 200 W metal arc lamp (Prior Scientific, UK) and an 60x objective lens and Volocity 3.6.1 software (Improvision). Scoring was carried out using exported tiff images for each individual condition within an experiment on at least 10 separate fields of view containing between 200–300 cells in total. Cells were scored positive for foci if they contained >5 discrete foci per nucleus, and the mean percentage of positive cells from at least 3 independent experiments were then calculated and plotted along with their respective standard errors of the means (SEM).

### Quantitative PCR

RNA was extracted from cells using a Qiagen RNEasy Plus Kit and quantified on a Nanodrop spectrophotometer. Equal quantities of RNA (2 μg) were then reverse transcribed using an Applied Biosystems High Capacity RNA- cDNA kit as per the manufacturer's instructions. Resulting cDNAs were appropriately diluted into 10 μl Taqman reactions in triplicate using an Applied Biosystems Taqman Gene Expression Master Mix and Applied Biosystems 7900HT Thermal cycler using the manufacturer's recommended conditions. Resulting data was subsequently analysed using Applied Biosystems SDS 2.4 software to calculate respective Dct values and relative gene expression levels. SYBER Green RT-PCR probes used were CDK18; For_TTCTCCCAACAGACAGCGG and Rev_GCAGCTGGAACATGAAAATCTTG, GAPDH; For_CTGGTAAAGTGGATTGTTGCCAT and Rev_TGGAATCATATTGGAACATGTAAACC, and TMBIM6 (TEGT) as previously described [42].

### Protein extraction and western blotting

Cell lysates were prepared in RIPA buffer, (50 mM Tris-HCL pH 8.0, 150 mM NaCl, 1% (v/v) NP40, 0.1% (w/v) SDS and 0.5% (w/v) sodium deoxycholate, supplemented with protease inhibitors tablets and Phostop tablets) for 30 minutes on ice. Resulting lysates were clarified at 16,000 × g for 20 minutes. Gel electrophoresis was performed using the NuPAGE system (Invitrogen). Briefly, samples were resolved on 4–12% Bis-Tris gels in MOPS buffer, transferred to a PVDF membrane. Membranes were incubated with antibodies diluted in PBS supplemented with 5% milk protein and incubated, with agitation, overnight at 4° C. Membranes were washed in PBS for 3 × 5 minutes and incubated in appropriate secondary antibodies for 1 hour at room temperature. The antibodies used for immunoblotting were as follows: anti-CDK18 (Santa Cruz Biotechnology: sc-176), anti-pCDK substrate ([K/H]pSP) MultiMab rabbit Ab mix (Cell Signalling), anti-CHK1 (Sigma Aldrich), anti-CHK1 pS317 (Cell Signaling), anti-RAD9 (Bethyl laboratories: A300–800A), anti-RPA2 (Calbiochem), anti-RPA2 pT21 (Abcam), anti-RPA2 pS4/8 (Bethyl Laboratories), anti-KAP1 (Bethyl Laboratories), anti-KAP1 pS824 (Bethyl Laboratories), anti-ATR (Santa Cruz Biotechnology), anti-Actin (Abcam), HRP-secondary antibodies (DAKO) and Alexa-Fluor antibodies (Invitrogen).

### Immunoprecipitation

For endogenous RAD9 immunoprecipitation, 2 mg of a whole-cell protein extract was added to 30 μl of pre-washed Protein G fast flow bead slurry (Calbiochem) together with 5 μg of mouse monoclonal antibody (ab150747 or sc-166457) per mg of whole cell extract, or no antibody for the bead only control, and incubated on a rotating wheel for 16 hours at 4° C. Beads were pelleted and washed several times for 15 mins each wash in 1 ml of TGN buffer (150 mM NaCl, 10% glycerol and 1% Tween-20 supplemented with Benzonase). Pelleted beads were then boiled in 2× LDS buffer supplemented with 3 mM DTT prior to SDS-PAGE and subsequent western blotting.

### Cell growth and cell cycle analyses

For cell growth assays, Cells were plated at low densities in 8 replicates within 96-well plates. After the indicated times, MTT reagent was added to the cells at a final concentration of 1 mg/ml, and these were incubated at 37° C for 3 hrs. The media was removed and 200 μl DMSO added to each well to solubilise the formazan product. The absorbance of the resulting product was assessed by quantifying optical density at 540 nm using a spectrophotometric microtitre plate reader. For cell cycle analyses, cell cultures were collected with their culture media and pelleted. Cell pellets were washed in PBS and fixed overnight in 70% Ethanol at –20° C. Fixed samples were washed 3 times in PBS before treatment with 5 μg of DNAse1 followed by the addition of 300 μl of Propidium Iodide (50 μg/ml) to each sample. FACS acquisition was carried out using a FACS-Calibur (Becton-Dickinson) and analysed by FlowJo (Tree Star, Inc). 10,000 live cells were gated and quantified for each sample.

### Clonogenic survival assays

Between 200–2000 cells were plated into 6-well plates fours hours prior to drug treatments to allow cells to attach. When colonies could be observed in the control plates (10–14 days post-treatment), cells were fixed and stained with methylene blue in methanol (4 g/L), and colonies consisting of more than 50 cells were subsequently scored. Surviving fractions (SF) were calculated based on the plating efficiency (fraction of colonies formed in untreated plates) for each cell population/treatment as; SF = (No. cells counted)/(No. cells plated × plating efficiency).

### Immunohistochemistry of cell-based sections

For antibody optimisation experiments, MDA-MB-231 and MCF-7 cells were transfected with either control or CDK18 siRNA for 72 hours as previously described [10]. Cells were then washed in PBS, trypsinised, pelleted and re-suspended in 1% molten agarose before being allowed to cool and set in a 1.5 ml eppendorf tube. The resulting cell pellets were then fixed in formalin, embedded in paraffin and 3 μm sections were mounted onto microscope slides for subsequent staining. Slides were imaged using a Leica Leitz DMRB microscope and captured with an Osteomeasure XP system at a magnification of x20.

### Commercial tissue microarray staining and scoring

Commercial breast cancer tissue microarrays (TMAs) were purchased from US Biomax Inc. and comprised of 366 readable samples, which included 334 breast cancers comprising of 292 invasive ductal carcinomas, 18 invasive intraductal, 12 invasive lobular, 4 cystosarcoma phyllodes, 4 medullary, and 4 apocrine carcinomas. The remaining samples included 20 normal breast tissue, and 18 cancer adjacent tissue samples which served as controls. For tissue microarray cores: formalin fixed paraffin embedded (FFPE) samples were deparaffinised in 2 xylene baths for 5–10 minutes each. Slides were then hydrated through descending grades of ethanol (100% to 70%) for 3 minutes each. After washing the slides in running water for 5 minutes, the sections were then immersed in sodium citrate at pH 6 (Sigma) and microwaved for 3 minutes at high temperature and 7 minutes at low temperature for antigen retrieval. The slides were allowed to cool in solution and then washed in distilled water for 15 minutes. Sections were then soaked in 3% hydrogen peroxide in methanol at 37° C for 30 minutes to block endogenous peroxidase activity. Following 3 washes in distilled water, slides were then incubated for 60 minutes in 10 % normal goat serum (Vector Laboratories) and 10% casein in PBS to block non-specific binding sites and then probed with the relevant primary antibodies prepared in PBS containing 2% sera and 2% casein overnight at 4° C. Next day, slides were rinsed in PBS and then incubated for 60 minutes at room temperature in biotinylated goat anti-rabbit secondary antibody (Vector Laboratories) prepared at 1:200 in PBS containing 2% sera and 2% casein. The antigen-antibody complexes were detected using the Vectastain Elite ABC kit (Vector Laboratories) according to manufacturer's protocol, followed by staining with 3,3’-diaminobenzidin (DAB) peroxidase substrate kit, (Vector Laboratories). Sections were then counterstained in Gill's haematoxylin and dehydrated in ascending grades of ethanol before clearing in xylene and mounting under a coverslip using DPX mountant (Fisher). For each IHC staining experiment, FFPE sections of CDK18-proficient and deficient cells (see Supplementary Figure 1A and 1B) were used as positive and negative controls respectively. An additional negative control consisted of omission of the primary antibody. Stained TMA slides were scanned using an Aperio ScanScope CS digital scanner and resulting images analysed by Aperio ImageScope viewing software (v11.1). Scoring of CDK18 staining was carried out using a 4 scale scoring system of 0; no/negligible staining, +1; weak/incomplete staining, 2; moderate staining, and +3; strong staining across the tissue section. Scoring of FFPE sections in the Nottingham cohort (see below) was carried out by two independent people and all sections were verified for tumour grade by a consultant pathologist. The resulting data was dichotomised using X-tiles software as well at Median/Mean depending on whether the data was parametric or non-parametric.

### Analysis of METABRIC datasets

X-tile (version 3.6.1) was used to identify a cut-off in CDK18 mRNA expression values to divide the population in to high/low mRNA expressing subgroups prior to analysis. The Chi-square test was used for testing association between categorical variables and a multivariate Cox model was fitted to the data using as endpoint breast cancer specific death. Cumulative survival probabilities were estimated using the Kaplan–Meier method.

### Patient study

The study was performed in a consecutive series of 1650 patients with primary invasive breast carcinomas who were diagnosed between 1986 and 1999 and entered into the Nottingham Tenovus Primary Breast Carcinoma series [18–21]. All patients were treated uniformly in a single institution and have been investigated in a wide range of biomarker studies [18–21]. Supplementary Table 2 summarises patient demographics. Supplementary treatment data summarizes various adjuvant treatments received by patients within this cohort. We also evaluated an independent series of 281 ER-α negative invasive breast cancers diagnosed and managed at the Nottingham University Hospitals between 1999 and 2007 [43]. All patients were primarily treated with surgery, followed by radiotherapy and anthracycline chemotherapy. The characteristics of this cohort are summarised in Supplementary Table 3. The Reporting Recommendations for Tumour Marker Prognostic Studies (REMARK) criteria, recommended by McShane and colleagues [44], were followed throughout this study. Breast cancer TMAs from these patients were constructed with 2 replicate 0.6 mm cores from the centre and periphery of the tumours. The TMAs were immunohistochemically profiled for XRCC1 and other biological antibodies as previously described [43]. Immunohistochemical staining for CDK18 was performed using the Bond Max automated staining machine and Leica Bond Refine Detection kit (DS9800) according to manufacturer instructions (Leica Microsystems). Pre-treatment of TMA sections was performed with citrate buffer (pH 6.0) antigen for 20 minutes. TMA sections were incubated for 15 minutes at room temperature with 1:100 anti-CDK18 mouse monoclonal antibody. HER2 expression was assessed according to the new ASCO/CAP guidelines using IHC and fluorescence *in situ* hybridisation (FISH). To validate the use of TMAs for immunophenotyping, full-face sections of 40 cases were stained and protein expression levels of the different antibodies were compared. The concordance between TMAs and full-face sections was deemed excellent (k = 0.8). Positive (normal kidney and normal liver tissue) and negative (by omission of the primary antibody and IgG-matched serum) controls were included in each batch of IHC experiments. Stained TMAs were evaluated by three specialist pathologists blinded to the clinicopathological characteristics of patients, in two different settings. There was excellent intra and inter-observer agreements (*k* > 0.8; Cohen's κ and multi-rater κ tests, respectively). Whole field inspection of the core was scored and intensities of nuclear staining were grouped as follows: 0 = no staining, 1 = weak staining, 2 = moderate staining, 3 = strong staining. The percentage of each category was estimated (0–100%). H-score (range 0–300) was calculated by multiplying intensity of staining and percentage staining as previously described (30–33). Low/negative CDK18 expression was defined by mean of H-score of ≤100. Not all cores within the TMA were suitable for IHC analysis due to missing cores or absence of tumour cells. This work was approved by Nottingham Research Ethics Committee.

### Statistical analyses

For IHC studies on commercial TMAs and Nottingham breast cancer cohort; data analysis was performed using SPSS (SPSS, version 17 Chicago, IL). Where appropriate, Pearson's Chi-square, Fisher's exact, Student's t and ANOVA one-way tests were used. Cumulative survival probabilities were estimated using the Kaplan–Meier method, and differences between survival rates were tested for significance using the log-rank test. Multivariate analysis for survival was performed using the Cox proportional hazard model. The proportional hazards assumption was tested using standard log-log plots. Hazard ratios (HR) and 95% confidence intervals (95% CI) were estimated for each variable. All tests were two-sided with a 95% CI and a *p* value < 0.05 considered significant. For multiple comparisons, *p* values were adjusted according to Holm-Bonferroni correction method. For study power calculations; sample size and effect size were determined using PASS (NCSS, version 11, USA). Cox regression of the log hazard ratio on CDK18 covariant (SD = 0.73), based on a sample of 1650 observations, achieves 90% power at the 0.05 significant level to detect a small regression coefficient equal to 0.25 and 0.28 for risk of recurrence and death, respectively. The sample size was adjusted since a multiple regression of the variable of interest on the other covariates in the Cox regression is expected to have an R squared of 0.3. The sample size was adjusted for recurrence and death event rate of 0.40 and 0.30 respectively. Statistically significant differences between quantified variables within parental MDA-MB-231 and CDK18 activation clone cell populations was confirmed using a 2-tailed *t*-test, assuming equal variances and are presented on figures as ^*^=*p* ≤ 0.05, ^**^= *p* ≤ 0.01.

## SUPPLEMENTARY MATERIALS FIGURES AND TABLES



## References

[1] Eccles SA, Aboagye EO, Ali S, Anderson AS, Armes J, Berditchevski F, Blaydes JP, Brennan K, Brown NJ, Bryant HE, Bundred NJ, Burchell JM, Campbell AM (2013). Critical research gaps and translational priorities for the successful prevention and treatment of breast cancer. Breast Cancer Res.

[2] Ali R, Rakha EA, Madhusudan S, Bryant HE (2017). DNA damage repair in breast cancer and its therapeutic implications. Pathology.

[3] Malumbres M (2014). Cyclin-dependent kinases. Genome Biol.

[4] Malumbres M, Barbacid M (2001). To cycle or not to cycle: a critical decision in cancer. Nat Rev Cancer.

[5] Whittaker SR, Mallinger A, Workman P, Clarke PA (2017). Inhibitors of cyclin-dependent kinases as cancer therapeutics. Pharmacol Ther.

[6] Kim ES, Scott LJ (2017). Palbociclib: A Review in HR-Positive, HER2-Negative, Advanced or Metastatic Breast Cancer. Target Oncol.

[7] Rocca A, Schirone A, Maltoni R, Bravaccini S, Cecconetto L, Farolfi A, Bronte G, Andreis D (2017). Progress with palbociclib in breast cancer: latest evidence and clinical considerations. Ther Adv Med Oncol.

[8] Vijayaraghavan S, Moulder S, Keyomarsi K, Layman RM (2018). Inhibiting CDK in Cancer Therapy: Current Evidence and Future Directions. Target Oncol.

[9] Miller SM, Goulet DR, Johnson GL (2017). Targeting the Breast Cancer Kinome. J Cell Physiol.

[10] Barone G, Staples CJ, Ganesh A, Patterson KW, Bryne DP, Myers KN, Patil AA, Eyers CE, Maslen S, Skehel JM, Eyers PA, Collis SJ (2016). Human CDK18 promotes replication stress signaling and genome stability. Nucleic Acids Res.

[11] Macheret M, Halazonetis TD (2015). DNA replication stress as a hallmark of cancer. Annu Rev Pathol.

[12] Gaillard H, García-Muse T, Aguilera A (2015). Replication stress and cancer. Nat Rev Cancer.

[13] Dobbelstein M, Sørensen CS (2015). Exploiting replicative stress to treat cancer. Nat Rev Drug Discov.

[14] Toledo L, Neelsen KJ, Lukas J (2017). Replication Catastrophe: When a Checkpoint Fails because of Exhaustion. Mol Cell.

[15] Gilad O, Nabet BY, Ragland RL, Schoppy DW, Smith KD, Durham AC, Brown EJ (2010). Combining ATR suppression with oncogenic Ras synergistically increases genomic instability, causing synthetic lethality or tumorigenesis in a dosage-dependent manner. Cancer Res.

[16] Curtis C, Shah SP, Chin SF, Turashvili G, Rueda OM, Dunning MJ, Speed D, Lynch AG, Samarajiwa S, Yuan Y, Gräf S, Ha G, Haffari G, METABRIC Group (2012). The genomic and transcriptomic architecture of 2,000 breast tumours reveals novel subgroups. Nature.

[17] Jia Y, Chen L, Jia Q, Dou X, Xu N, Liao DJ (2016). The well-accepted notion that gene amplification contributes to increased expression still remains, after all these years, a reasonable but unproven assumption. J Carcinog.

[18] Alsubhi N, Middleton F, Abdel-Fatah TM, Stephens P, Doherty R, Arora A, Moseley PM, Chan SY, Aleskandarany MA, Green AR, Rakha EA, Ellis IO, Martin SG (2016). Chk1 phosphorylated at serine345 is a predictor of early local recurrence and radio-resistance in breast cancer. Mol Oncol.

[19] Arora A, Abdel-Fatah TM, Agarwal D, Doherty R, Croteau DL, Moseley PM, Hameed K, Green A, Aleskandarany MA, Rakha EA, Patterson K, Ball G, Chan SY (2016). Clinicopathological and prognostic significance of RECQL5 helicase expression in breast cancers. Carcinogenesis.

[20] Arora A, Abdel-Fatah TM, Agarwal D, Doherty R, Moseley PM, Aleskandarany MA, Green AR, Ball G, Alshareeda AT, Rakha EA, Chan SY, Ellis IO, Madhusudan S (2015). Transcriptomic and Protein Expression Analysis Reveals Clinicopathological Significance of Bloom Syndrome Helicase (BLM) in Breast Cancer. Mol Cancer Ther.

[21] Arora A, Parvathaneni S, Aleskandarany MA, Agarwal D, Ali R, Abdel-Fatah T, Green AR, Ball GR, Rakha EA, Ellis IO, Sharma S, Madhusudan S (2017). Clinicopathological and Functional Significance of RECQL1 Helicase in Sporadic Breast Cancers. Mol Cancer Ther.

[22] Holliday DL, Speirs V (2011). Choosing the right cell line for breast cancer research. Breast Cancer Res.

[23] Cleaver JE (2011). γH2Ax: biomarker of damage or functional participant in DNA repair “all that glitters is not gold!”. Photochem Photobiol.

[24] Lee J, Kumagai A, Dunphy WG (2007). The Rad9-Hus1-Rad1 checkpoint clamp regulates interaction of TopBP1 with ATR. J Biol Chem.

[25] St Onge RP, Besley BD, Pelley JL, Davey S (2003). A role for the phosphorylation of hRad9 in checkpoint signaling. J Biol Chem.

[26] Ueda S, Takeishi Y, Ohashi E, Tsurimoto T (2012). Two serine phosphorylation sites in the C-terminus of Rad9 are critical for 9-1-1 binding to TopBP1 and activation of the DNA damage checkpoint response in HeLa cells. Genes Cells.

[27] Bartkova J, Rezaei N, Liontos M, Karakaidos P, Kletsas D, Issaeva N, Vassiliou LV, Kolettas E, Niforou K, Zoumpourlis VC, Takaoka M, Nakagawa H, Tort F (2006). Oncogene-induced senescence is part of the tumorigenesis barrier imposed by DNA damage checkpoints. Nature.

[28] Di Micco R, Fumagalli M, Cicalese A, Piccinin S, Gasparini P, Luise C, Schurra C, Garre' M, Nuciforo PG, Bensimon A, Maestro R, Pelicci PG, d’Adda di Fagagna F (2006). Oncogene-induced senescence is a DNA damage response triggered by DNA hyper-replication. Nature.

[29] Hanahan D, Weinberg RA (2011). Hallmarks of cancer: the next generation. Cell.

[30] Hills SA, Diffley JF (2014). DNA replication and oncogene-induced replicative stress. Curr Biol.

[31] Bartkova J, Horejsí Z, Koed K, Krämer A, Tort F, Zieger K, Guldberg P, Sehested M, Nesland JM, Lukas C, Ørntoft T, Lukas J, Bartek J (2005). DNA damage response as a candidate anti-cancer barrier in early human tumorigenesis. Nature.

[32] Gorgoulis VG, Vassiliou LV, Karakaidos P, Zacharatos P, Kotsinas A, Liloglou T, Venere M, Ditullio RA, Kastrinakis NG, Levy B, Kletsas D, Yoneta A, Herlyn M (2005). Activation of the DNA damage checkpoint and genomic instability in human precancerous lesions. Nature.

[33] Di Benedetto A, Ercolani C, Mottolese M, Sperati F, Pizzuti L, Vici P, Terrenato I, Shaaban AM, Humphries MP, Di Lauro L, Barba M, Vitale I, Ciliberto G (2017). Analysis of the ATR-Chk1 and ATM-Chk2 pathways in male breast cancer revealed the prognostic significance of ATR expression. Sci Rep.

[34] Koschmann C, Farooqui Z, Kasaian K, Cao X, Zamler D, Stallard S, Venneti S, Hervey-Jumper S, Garton H, Muraszko K, Franchi L, Robertson PL, Leonard M (2017). Multi-focal sequencing of a diffuse intrinsic pontine glioma establishes PTEN loss as an early event. NPJ Precis Oncol.

[35] Zeman MK, Cimprich KA (2014). Causes and consequences of replication stress. Nat Cell Biol.

[36] Buisson R, Boisvert JL, Benes CH, Zou L (2015). Distinct but Concerted Roles of ATR, DNA-PK, and Chk1 in Countering Replication Stress during S Phase. Mol Cell.

[37] Ying S, Chen Z, Medhurst AL, Neal JA, Bao Z, Mortusewicz O, McGouran J, Song X, Shen H, Hamdy FC, Kessler BM, Meek K, Helleday T (2016). DNA-PKcs and PARP1 Bind to Unresected Stalled DNA Replication Forks Where They Recruit XRCC1 to Mediate Repair. Cancer Res.

[38] Caldon CE (2014). Estrogen signaling and the DNA damage response in hormone dependent breast cancers. Front Oncol.

[39] Kanu N, Cerone MA, Goh G, Zalmas LP, Bartkova J, Dietzen M, McGranahan N, Rogers R, Law EK, Gromova I, Kschischo M, Walton MI, Rossanese OW (2016). DNA replication stress mediates APOBEC3 family mutagenesis in breast cancer. Genome Biol.

[40] Canavese M, Santo L, Raje N (2012). Cyclin dependent kinases in cancer: potential for therapeutic intervention. Cancer Biol Ther.

[41] Staples CJ, Myers KN, Beveridge RD, Patil AA, Howard AE, Barone G, Lee AJ, Swanton C, Howell M, Maslen S, Skehel JM, Boulton SJ, Collis SJ (2014). Ccdc13 is a novel human centriolar satellite protein required for ciliogenesis and genome stability. J Cell Sci.

[42] Andersen CL, Jensen JL, Ørntoft TF (2004). Normalization of real-time quantitative reverse transcription-PCR data: a model-based variance estimation approach to identify genes suited for normalization, applied to bladder and colon cancer data sets. Cancer Res.

[43] Abdel-Fatah TM, Arora A, Moseley PM, Perry C, Rakha EA, Green AR, Chan SY, Ellis IO, Madhusudan S (2015). DNA repair prognostic index modelling reveals an essential role for base excision repair in influencing clinical outcomes in ER negative and triple negative breast cancers. Oncotarget.

[44] McShane LM, Altman DG, Sauerbrei W, Taube SE, Gion M, Clark GM, Statistics Subcommittee of the NCI-EORTC Working Group on Cancer Diagnostics (2005). REporting recommendations for tumour MARKer prognostic studies (REMARK). Eur J Cancer.

[45] Szász AM, Lánczky A, Nagy Á, Förster S, Hark K, Green JE, Boussioutas A, Busuttil R, Szabó A, Győrffy B (2016). Cross-validation of survival associated biomarkers in gastric cancer using transcriptomic data of 1,065 patients. Oncotarget.

