# Supplementary material for: The relationship of CDK18 expression in breast cancer to clinicopathological parameters and therapeutic response
Source: Oncotarget. 2018 Jun 29;9(50):29508–24. doi: 10.18632/oncotarget.25686 (PMC6047673; doi:10.18632/oncotarget.25686)
Supplement: Supplementary file 1 [file oncotarget-09-29508-s001.pdf]

The relationship of CDK18 expression in breast cancer to clinicopathological parameters and therapeutic response

SUPPLEMENTARY MATERIALS

Supplementary patient treatment data for Nottingham Tenovus BC cohort

Patients received standard surgery (mastectomy or wide local excision) with radiotherapy. Prior to 1989, patients did not receive systemic adjuvant treatment (AT). After 1989, AT was scheduled based on prognostic and predictive factor status, including NPI, oestrogen receptor-α (ER-α) status, and menopausal status. Patients with NPI scores of <3.4 (low risk) did not receive AT. In pre-menopausal patients with NPI scores of ≥3.4 (high risk), classical Cyclophosphamide, Methotrexate, and 5-Flourouracil (CMF) chemotherapy was given; patients with ER-α positive tumours were also offered HT. Postmenopausal patients with NPI scores of ≥3.4 and ER-α positivity were offered HT, while ER-α negative patients received classical CMF chemotherapy. Median follow up was 111 months (range 1 to 233 months). Survival data, including overall survival, disease-free survival (DFS), and development of loco-regional and distant metastases (DM), was maintained on a prospective basis. DFS was defined as the number of months from diagnosis to the occurrence of local recurrence, local lymph node (LN) relapse or DM relapse. Breast cancer specific survival (BCSS) was defined as the number of months from diagnosis to the occurrence of BC related-death. Local recurrence free survival (LRS) was defined the number of months from diagnosis to the occurrence of local recurrence. DM-free survival was defined as the number of months from diagnosis to the occurrence of DM relapse. Survival was censored if the patient was still alive at the time of analysis, lost to follow-up, or died from other causes.

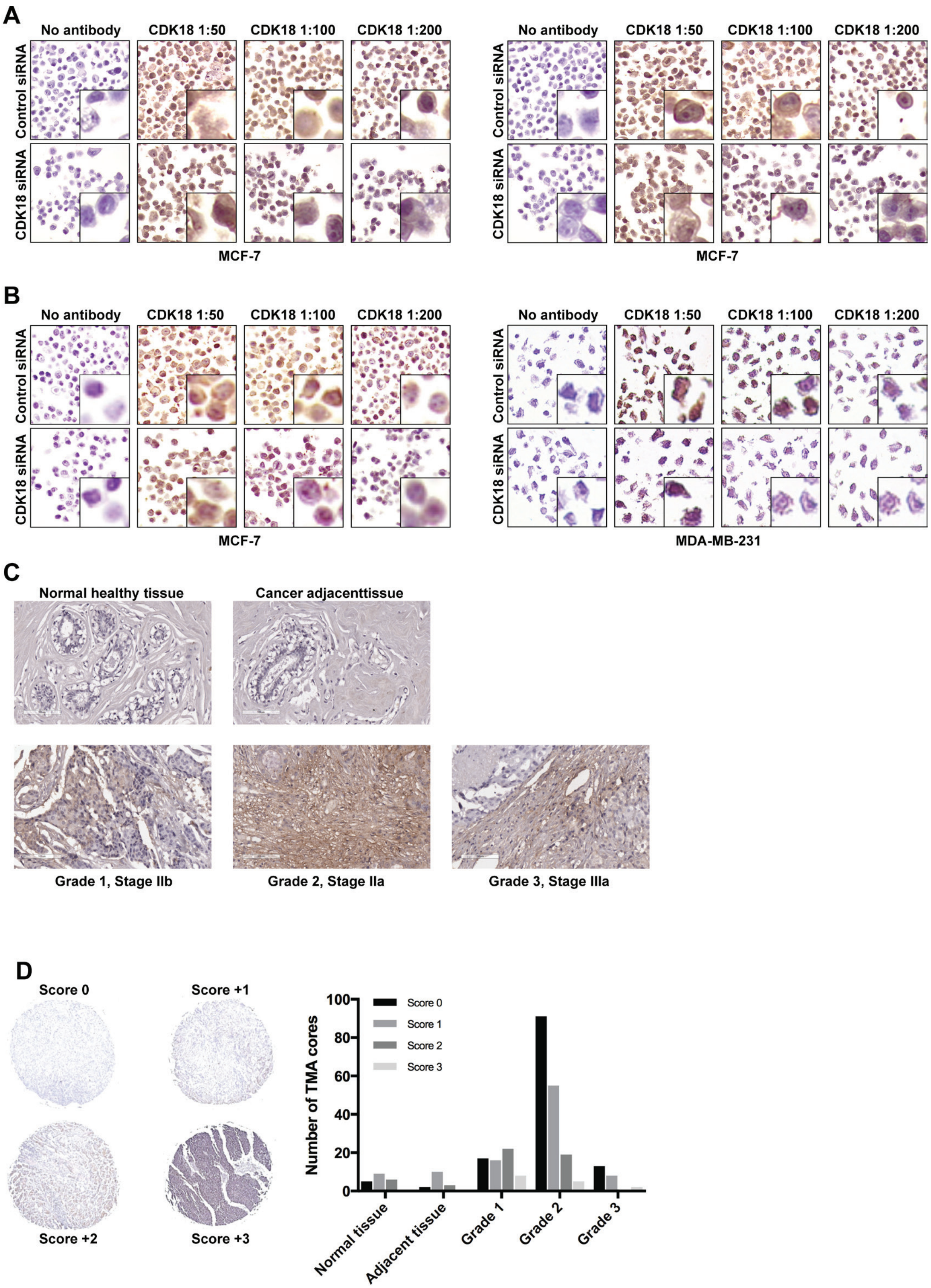

Supplementary Figure 1: (**A**) Representative images of IHC staining optimisation for CDK18 in MCF-7 cells transfected with control (non-targeting) or previously validated CDK18 siRNA[10] as indicated. The images in the left and right panels represent two independent experiments. (**B**) As in A, but in MCF-7 (left panel) and MDA-MB-231 cells (right panel) from an additional experimental repeat. (**C**) Representative images of CDK18 IHC staining (at 1:100 based on the optimisation data above) in commercial breast cancer TMAs. (**D**) Scoring of CDK18 IHC staining in commercial breast cancer TMAs. Left panel; representative images to highlight the scoring system used. Right panel; quantification of CDK18 scoring across the various normal and tumour samples within the TMAs.

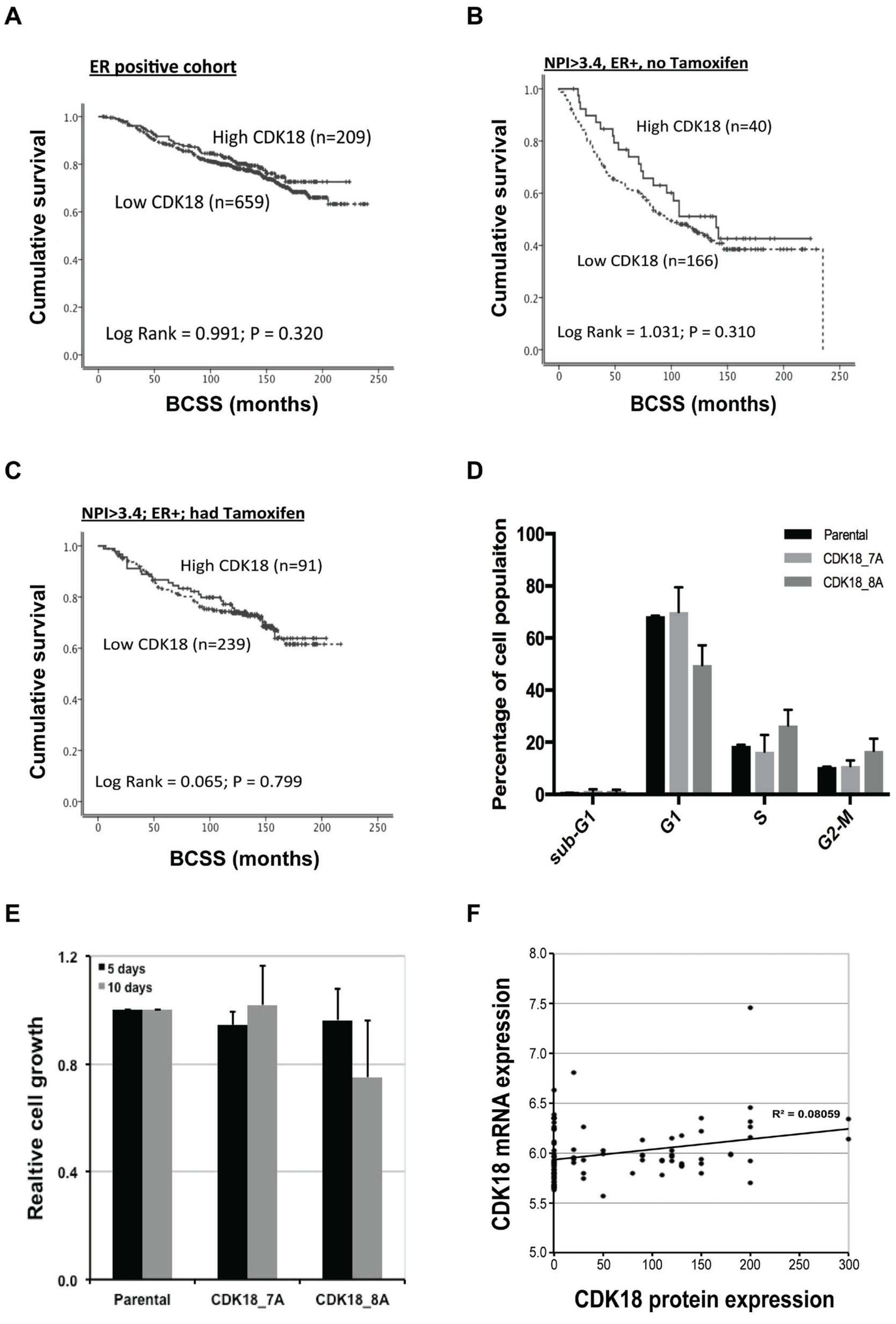

Supplementary Figure 2: (**A–C**) Kaplan-Meier survival curves for CDK18 protein expression (above or below median) plotted against breast cancer specific survival for ER+ tumours within the Nottingham Tenovus breast cancer cohort stratified using the indicated clinicopathological parameters. (**D**) Quantification of cell cycle phases in parental MDA-MB-231 cells and CDK18 activation clones as indicated. Data shown represents the mean derived from at least two independent experiments with their respective SEMs. (**E**) Growth of CRISPR CDK18 knockout and activation clones compared with parental MDA-MB-231 cells over 5-day or 10-day period as indicated. Data shown represents the mean derived from at least five independent experiments with their respective SEMs. (**F**) CDK18 mRNA and protein expression levels within individual tumours in the Nottingham Tenovus breast cancer cohort showing a lack of correlation between the two in 86 individual tumours.

Supplementary Table 1: Clinicopathological characteristics of the Nottingham Tenovus breast cancer cohort

Variable	*n*[*]	Cases	(%)
**Menopausal status**	1650		
Pre-menopausal		612	(37.0)
postmenopausal		1038	(63.0)
**Tumour Grade (NGS)**	1650		
G1		306	(18.5)
G2		531	(32.2)
G3		813	(49.3)
**Lymph node stage**	1650		
Negative		1056	(64.0)
Positive (1–3 nodes)		486	(29.5)
Positive (>3 nodes)		10	(6.5)
**Tumour size (cm)**	1650		
T1 a + b (<1.0)		187	(11.0)
T1 c (>1.0–2.0)		868	(53.0)
T2 (>2.0–5)		579	(35.0)
T3 (>5)		16	(1.0)
**Tumour type**	1650		
IDC-NST		941	(57)
Tubular		349	(21)
ILC		160	(10)
Medullary (typical/atypical)		41	(2.5)
Others		159	(9.5)
**NPI subgroups**	1650		
Excellent PG (2.08–2.40)	Low risk	207	(12.5)
Good PG (2.42–3.40)		331	(20.1)
Moderate I PG (3.42 to 4.4)	High risk	488	(29.6)
Moderate II PG (4.42 to 5.4)		395	(23.9)
Poor PG (5.42 to 6.4)		170	(10.3)
Very poor PG (6.5–6.8)		59	(3.6)
**Survival at 20 years**	1650		
Alive and well		1055	(64.0)
Dead from disease		468	(28.4)
Dead from other causes		127	(7.6)
**Adjuvant systemic therapy (AT)**			
No AT		665	(42.0)
Hormone therapy (HT)		642	(41.0)
Chemotherapy		307	(20.0)
Hormone + chemotherapy		46	(3.0)

Supplementary Table 2: Clinicopathological characteristics of validation set of ER negative breast cancer patient samples

Variable	*n*[*]	Cases	(%)
**Menopausal status**	252		
Pre-menopausal		122	(48.5)
postmenopausal		130	(51.5)
**Tumour Grade (NGS)**	252		
G1		1	(0.3)
G2		27	(10.6)
G3		224	(89.1)
**Lymph node stage**	252		
Negative		121	(48)
Positive (1–3 nodes)		86	(34)
Positive (>3 nodes)		45	(18)
Tumour size (cm)	252		
T1 a + b (≤1.0)		28	(11)
T1 c (>1.0–2.0)		106	(42)
T2 (>2.0–5)		103	(41)
T3 (>5)		15	(6)
**Tumour type**	252		
IDC-NST		224	(89.0)
Tubular		5	(2.0)
ILC		8	(3.0)
Medullary (typical/atypical)		5	(2.0)
Others		0	(4.0)
**NPI subgroups**	252		
Excellent PG (2.08–2.40)	Low risk	0	(0.0)
Good PG (2.42–3.40)		0	(0.0)
Moderate I PG (3.42 to 4.4)	High risk	111	(44.0)
Moderate II PG (4.42 to 5.4)		81	(32.0)
Poor PG (5.42 to 6.4)		38	(15.0)
Very poor PG (6.5–6.8)		22	(9.0)
**Survival at 5 years**	252		
Alive and well		176	(70.0)
Dead from disease		73	(29.0)
Dead from other causes		3	(1.0)

Supplementary Table 3: Clinicopathological characteristics of validation set of ER negative BC patient samples

Variable	*n**	Cases	(%)
**Menopausal status**	252		
Pre-menopausal		122	(48.5)
postmenopausal	130	(51.5)	
**Tumour Grade (NGS)**	252		
G1		1	(0.3)
G2		27	(10.6)
G3		224	(89.1)
**Lymph node stage**	252		
Negative		121	(48)
Positive (1–3 nodes)		86	(34)
Positive (>3 nodes)		45	(18)
**Tumour size (cm)**	252		
T1 a + b (≤1.0)		28	(11)
T1 c (>1.0–2.0)		106	(42)
T2 (>2.0–5)		103	(41)
T3 (>5)		15	(6)
**Tumour type**	252		
IDC-NST	224	(89.0)	
Tubular	5	(2.0)	
ILC	8	(3.0)	
Medullary (typical/atypical)	5	(2.0)	
Others	0	(4.0)	
**NPI subgroups**	252		
Excellent PG (2.08–2.40)	Low risk	0	(0.0)
Good PG (2.42–3.40)		0	(0.0)
Moderate I PG (3.42 to 4.4)	High risk	111	(44.0)
Moderate II PG (4.42 to 5.4)		81	(32.0)
Poor PG (5.42 to 6.4)		38	(15.0)
Very poor PG (6.5–6.8)		22	(9.0)
**Survival at 5 years**	252		
Alive and well		176	(70.0)
Dead from disease		73	(29.0)
Dead from other causes		3	(1.0)
